# Supplementary material for: Dynamic Changes in Human Milk Oligosaccharides in Chinese Population: A Systematic Review and Meta-Analysis
Source: Nutrients. 2021 Aug 24;13(9):2912. doi: 10.3390/nu13092912 (PMC8464947; doi:10.3390/nu13092912)
Supplement: Supplementary file 1 [file nutrients-13-02912-s001.zip › nutrients-1346936-supplementary.pdf]

Table S1. Inclusion and exclusion criteria of article selection

Parameter	Inclusion criteria	Exclusion criteria
**Population**	Chinese population; healthy mothers with healthy neonates.	Non-Chinese populations; non-human; mothers or infants with defined diseases or disorders; population without defined location in China.
**Intervention**	N/A	N/A
**Comparator**	N/A	N/A
**Outcomes**	Absolute concentrations of a single HMOs<br>Mean values±Standard Deviation<br>Single HMOs concentration values with n≥2 at given lactation period or secretor status.	Lactation stages were not specified<br>Milk samples were pooled together before component analysis<br>levels of individual HMOs not reported<br>Concentrations of mixtures of HMOs<br>Median values or relative concentrations.
**Study design**	Original articles from peer-reviewed journals; articles in Chinese from "core" Chinese journals or thesis.	Review articles; abstracts; articles without access to full texts; for articles that reported the same study (e.g., theses and journal articles that reported the same study), the theses which reported more comprehensive data were included.

Table S2. Quality assessment of included studies

Question	[4]	[14]	[29]	[30]	[31]	[32]	[33]	[34]
**Q1:** Did the authors clearly describe the period of investigation?	Y[1]	Y	Y	Y	Y	Y	Y	Y
**Q2:** Did the authors clearly describe the number of mothers recruited in the study?	Y	Y	N[2]	Y	N	Y	Y	Y
**Q3:** Did the authors describe clearly that the infants were full-term or pre-term?	Y	Y	Y	N	N	Y	Y	Y
**Q4:** Did the authors describe the delivery mode?	N	N	N	N	N	N	N	N
**Q5:** Did the authors describe clearly that the mothers and infants were healthy?	Y	Y	Y	Y	Y	Y	Y	Y
**Q6:** Was there dietary intervene the diet on the mothers in the study?	N	N	N	N	N	N	N	N
**Q7:** Did the authors report the secretor status or Lewis types of the samples?	N	Y	N	N	N	N	Y	N
**Q8:** Did the authors clearly describe the site where the human milk samples were collected?	Y	Y	Y	Y	Y	Y	Y	Y
**Q9:** Did the authors clearly describe the time of the day when human milk was collected, or all human milk were collected at the same time range of the day?	Y	Y	N	N	N	Y	Y	N
**Q10:** Did the authors describe clearly human milk was foremilk, hindmilk or full expression?	Y	Y	Y	N	N	Y	Y	N
**Q11:** Did the authors provide detailed determination method of fatty acids in human milk?	Y	Y	Y	Y	Y	Y	Y	Y
**Q12:** Did the authors describe funding sources (for-profit organizations, non-profit funding, or government funding) and conflicts of interest of the authors?	Y	N	Y	N	Y	Y	Y	Y

[1] Yes; [2] No.

Table S3. Blueprint of HMOs

Abbreviation	IUPAC-condensed nomenclature	Symbol Nomenclature for Glycans
**2′-FL**	Fuc(a1-2)Gal(b1-4)Glc	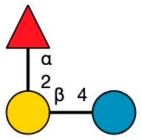
**3′-FL**	Gal(b1-4)[Fuc(a1-3)]Glc	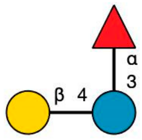
**3′-SL**	Neu5Ac(a2-3)Gal(b1-4)Glc	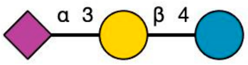
**6′-SL**	Neu5Ac(a2-6)Gal(b1-4)Glc	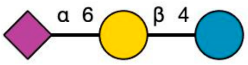
**LNT**	Gal(b1-3)GlcNAc(b1-3)Gal(b1-4)Glc	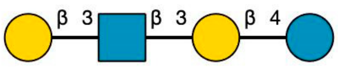
**LNnT**	Gal(b1-4)GlcNAc(b1-3)Gal(b1-4)Glc	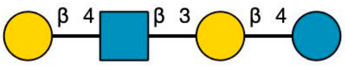
**LNDFH I**	Fuc(a1-2)Gal(b1-3)[Fuc(a1-4)]GlcNAc(b1-3)Gal(b1-4)Glc	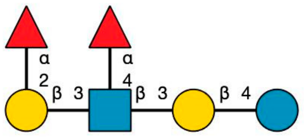

**LNDFH II**	Fuc(a1-4)[Gal(b1-3)]GlcNAc(b1-3)Gal(b1-4)[Fuc(a1-3)]Glc	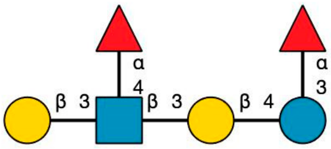
**DSLNT**	Neu5Ac(a2-3)Gal(b1-3)[Neu5Ac(a2-6)]Glc-NAc(b1-3)Gal(b1-4)Glc	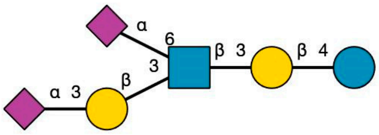
**LNFP I**	Fuc(a1-2)Gal(b1-3)GlcNAc(b1-3)Gal(b1-4)Glc	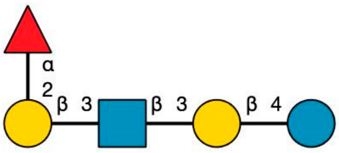
**LNFP II**	Fuc(a1-4)[Gal(b1-3)]GlcNAc(b1-3)Gal(b1-4)Glc	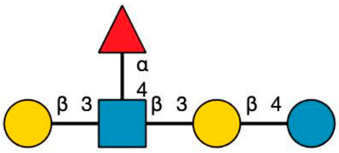
**LNFP III**	Fuc(a1-3)[Gal(b1-4)]GlcNAc(b1-3)Gal(b1-4)Glc	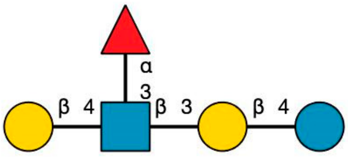
**LNFP V**	Gal(b1-3)GlcNAc(b1-3)Gal(b1-4)[Fuc(a1-3)]Glc	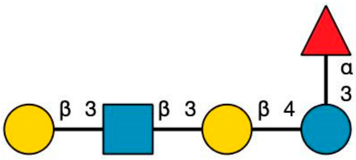
**LSTc**	Neu5Ac(a2-6)Gal(b1-4)GlcNAc(b1-3)Gal(b1-4)Glc	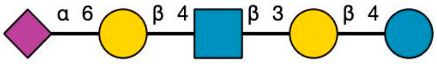

**Gal(Galactose)**	Gal	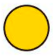
**Glc(Glucose)**	Glc	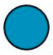
**GlcNAc(*N*-acety-glucosamine)**	GlcNAc	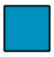
**Fuc(Fucose)**	Fuc	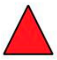
**Neu5Ac(*N*-acetylneuraminic acid)**	Neu5Ac	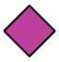

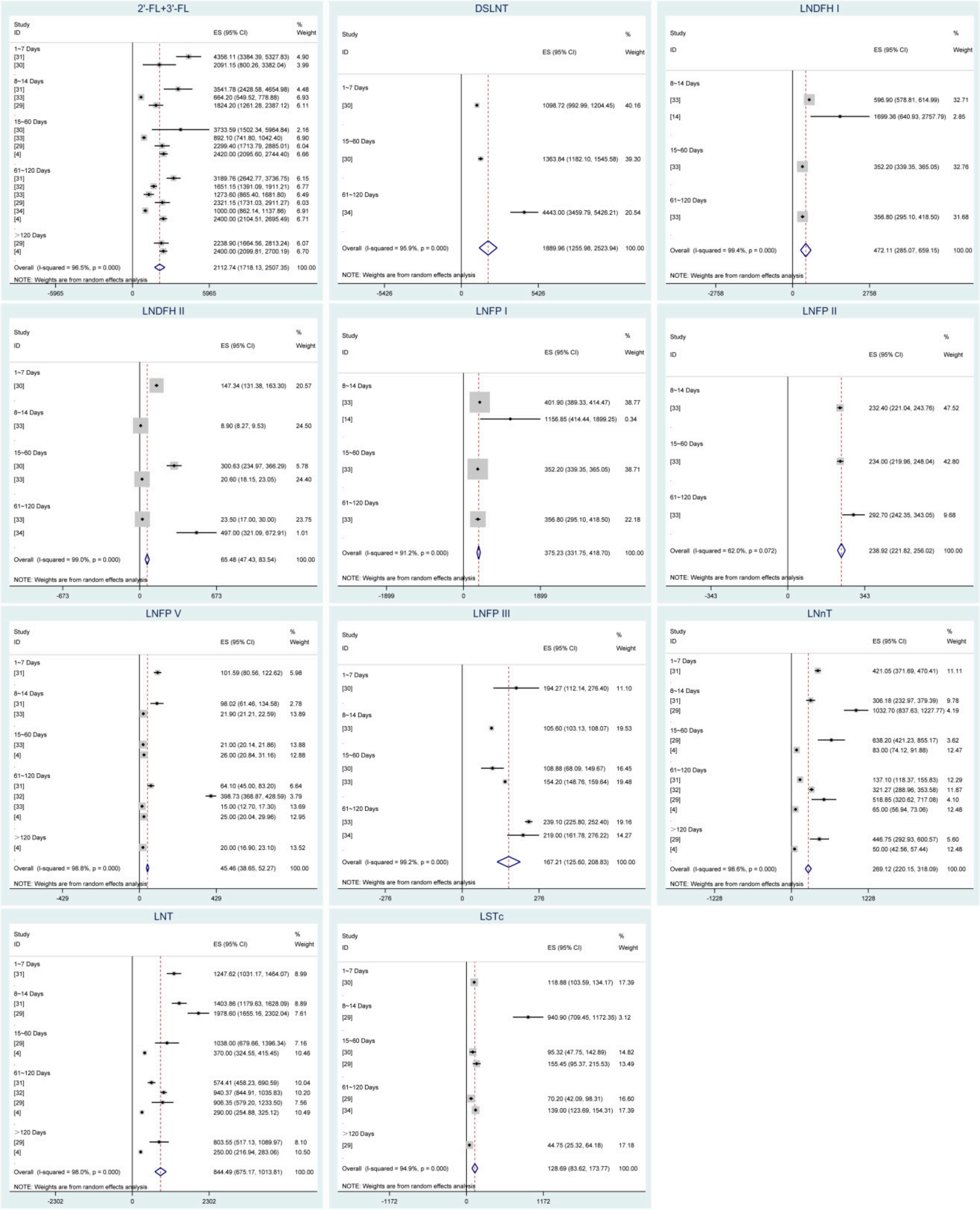

Figure S1. Forrest plots of HMO concentrations of included studies

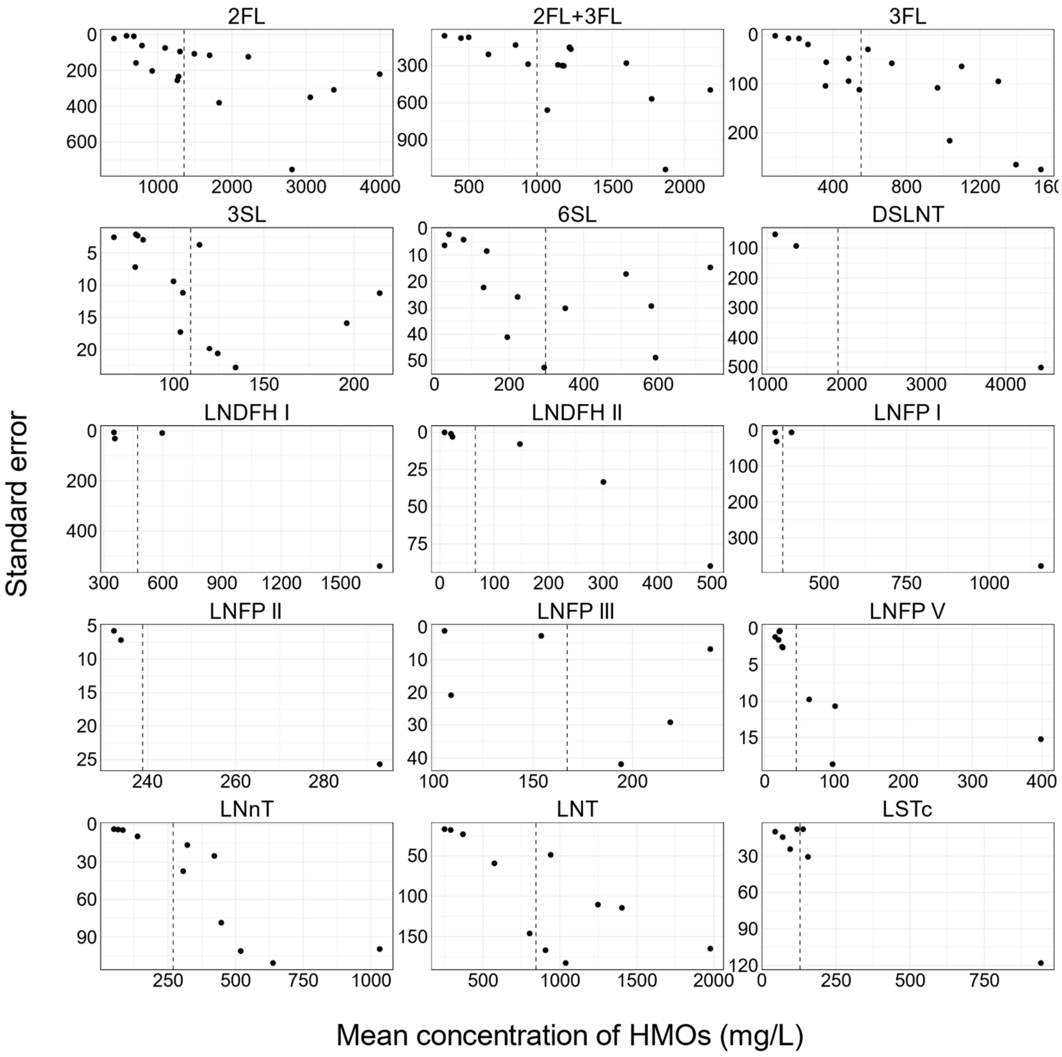

Figure S2. Publication biases of included studies
